# Does the Employability Paradox Exist or Not? An Inverted U-Shaped Model

**DOI:** 10.3389/fpsyg.2021.588793

**Published:** 2021-07-30

**Authors:** Haibo Yu, Changli Yan, Lu Zhang, Zhenhua Dong, Long Cheng, Xiaoming Zheng, Ziqian Zhao

**Affiliations:** ^1^School of Government, Beijing Normal University, Beijing, China; ^2^School of Business Administration, Ulsan National Institute of Science and Technology, Ulsan, South Korea; ^3^School of Business Administration, Shandong Women’s University, Jinan, China; ^4^Science and Technology Talent Exchange and Development Service Center, Ministry of Science and Technology, Beijing, China; ^5^School of Economics and Management, Tsinghua University, Beijing, China

**Keywords:** employability paradox, perceived employability, turnover intention, job performance, work seniority, the conservation of resource theory, the prospect theory, too much of a good thing effect

## Abstract

This paper’s purpose is to test the employability paradox by adopting a combined linear and non-linear approach based on the conservation of resource (COR) theory and the prospect theory and further to discuss it in two groups of employees with different seniority following the career timetable perspective. A total of 623 pairs of matched employee and manager surveys was collected from 27 Chinese enterprises in two waves. Hierarchical regression analysis was used to test the hypotheses. The results show no paradox that perceived employability promotes both an employee’s turnover intention and performance. Specifically, perceived employability has a significant inverted U-shaped effect on turnover intention but no direct influence on job performance. Seniority is a moderator, showing the curvilinear relationship only exhibits for employees with shorter work seniority (≤3 years), and a positive linear relationship between perceived employability and job performance only exists for employees with longer seniority (>3 years). This study emphasizes the value of employability for employers and proposes who is more suitable and what timetable should be followed for employability enhancement in practice. In addition, the study provides an enlightening finding of the inverted U-shaped relationship between perceived employability and turnover intention, applies the COR theory and the prospect theory to explain the non-linear relationship, validates the effect of too much of a good thing (TMGT), and negates the paradox from the perspective of the perceived general employability and career timetable.

## Introduction

The employability paradox has become a focal issue in recent years because the divergent views on its existence affect employers’ human resource practices (Rodrigues et al., 2020). The paradox refers to the phenomenon that employability has a positive effect on an employee’s performance but at the same time promotes turnover intention, which is considered a negative risk factor for organizations (De Grip et al., 2004; De Cuyper et al., 2011a; Nelissen et al., 2017). Previous studies have obtained conflicting results regarding the employability paradox. On one hand, evidence supports the paradox’s existence, because perceived employability can increase both performance and turnover intention (De Cuyper et al., 2011a). On the other hand, some researchers have claimed that the employability paradox is false by dividing employability into internal and external employability. These researchers argue that internal employability reduces turnover and job searching intensity and increases performance, whereas external employability has the opposite effect. Accordingly, the researchers criticize the paradox research relying on the general employability (De Cuyper and De Witte, 2011; De Vos et al., 2017; Nelissen et al., 2017).

However, researchers cannot reconcile past findings by simply separating the effects of internal vs. external employability. For example, prior studies have reported mixed findings for the relationship between perceived employability and turnover intention, including positive, negative, and null effect (Berntson et al., 2010; De Cuyper et al., 2011b; Acikgoz et al., 2016). From the internal vs. external employability perspective, the positive employability and turnover intention relationship may be attributable to external employability, whereas the negative relationship may be due to internal employability. However, past research has shown that internal and external employability tend to strongly correlate with each other (De Cuyper and De Witte, 2011; De Vos et al., 2017; Nelissen et al., 2017). In other words, a person is apt to have high-high or low-low internal and external employability, respectively. As such, the opposing effects of internal and external employability may cancel each other out, and it remains difficult to explain the mixed relationship by distinguishing internal and external employability. Therefore, scholars need to develop alternative theoretical perspectives to better understand the employability paradox. Research needs to clarify whether and under what conditions the employability paradox exists, which will inform the employability investment in practice.

This study’s main purpose is to provide an alternative explanation to address whether the paradox of employability exists. Using conservation of resource (COR) theory and prospect theory as lenses, we propose that there can be a combination of linear and non-linear relationships between perceived general employability and performance/turnover. In other words, we expect that there is an inverted U-shaped relationship between employability and turnover, whereas employability is positively related to performance. Furthermore, we consider employees’ seniority as an important boundary condition to the aforementioned relationships. On the basis of career timetable theory, we expect that seniority will diminish the employability-turnover relationship and magnify the employability-performance relationship.

### Employability and Employability Paradox

From the perspective of personal resources in COR theory (Hobfoll et al., 2018), employability is a type of personal resource in the workplace (Acikgoz et al., 2016). The current definition of employability is divided into two perspectives. The first definition focuses on factors that increase the likelihood of employment, including competencies (Van der Heijden et al., 2018), dispositions (Fugate and Kinicki, 2008), and social capital characteristics (Leana and Van Buren, 1999). The second definition, adopted in this study, is based on personal views on their employment results, which is defined as personal perception of the possibility of entering and maintaining the employment status (Berntson and Marklund, 2007; Rothwell and Arnold, 2007; Virga et al., 2017). This conceptualization considers employees’ perceptions of the possibility of achieving employment goals in the job market (De Cuyper et al., 2012), and it relies on employees’ subjective feelings after considering both objective personal factors and environmental factors. Compared with the first definition, the latter is more predictive of individual behavior (De Cuyper et al., 2011a).

The paradox of employability refers to the fact that improved employability can bring both positive impacts and negative risks to the organization (De Grip et al., 2004). The positive impact usually refers to improvement in employees’ performance and organizational behaviors, whereas the negative impact is reflected in increased turnover intention, job search intensity and counterproductive work behaviors or reduced emotional organizational commitment (De Cuyper et al., 2011a; De Cuyper and De Witte, 2011; Acikgoz et al., 2016; De Vos et al., 2017; Nelissen et al., 2017; Imam and Chambel, 2020; Rodrigues et al., 2020). In this study, we focus on the paradox that improvement in perceived employability brings about the increase in both performance and turnover intention. This paradox is mostly discussed in theory (e.g., De Grip et al., 2004; Van der Heijde and Van der Heijden, 2006), whereas there are few empirical tests. In the empirical field, except for De Cuyper et al. (2011a) and Imam and Chambel (2020) who found that employability can bring positive and negative results at the same time, the remaining studies weaken or negate the employability paradox from the perspective of perceived internal and external employability (De Cuyper and De Witte, 2011; De Vos et al., 2017; Nelissen et al., 2017; Rodrigues et al., 2020). For example, Nelissen et al. (2017) believed that to explain why employability has different effects on the same variable, it should be discussed from the perspectives of internal and external employability, respectively, because they may have opposite effects on the same outcome variable: the former reduces turnover and the latter promotes turnover. We recognize the value of this separate and detailed discussion but also insist on discussing the paradox’s significance from the perspective of perceived general employability for two reasons. First, theoretically, internal and external employability reflects the employees’ perception of the degrees of achieving employment goals, and they both come from the ability, personality, and social capital of individuals. As a comprehensive quality, high perceived employability is likely to cause individuals to perceive both high internal and external employability at the same time. In other words, in one particular person, we usually observe high or low perceived internal and external employability at the same time. To achieve a lower turnover intention, according to De Vos et al. (2017) and Nelissen et al. (2017), a person should have both low perceived external employability and high internal employability, but such a state of one high and one low is difficult to maintain. Empirical data also show a significant positive correlation between perceived internal and external employability (De Cuyper and De Witte, 2011; De Vos et al., 2017; Nelissen et al., 2017). Therefore, it remains difficult to explain the mixed results in previous studies that indicate the employability-turnover intention relationship can be positive, negative, or irrelevant by distinguishing between internal and external employability. In an individual, the negative and positive effects of internal and external employability with nearly same level (both high or both low) on turnover intention offset one another; therefore, forming a significant positive or negative correlation between the general employability and turnover intention by the integration effects of the two is difficult. Second, in practice, organizations are concerned about the existence of a general employability paradox in order to decide whether to increase their investment in employees’ employability. Employability investment mainly provides employees with up-to-date knowledge and a broader set of skills (De Grip et al., 2004; De Cuyper et al., 2011a), which improve their general employability. For example, Nelissen et al. (2017) believe that formal off-the-job training will improve the internal and external employability perceived by employees at the same time, that is, the general employability we emphasized. Therefore, from an organizational point of view, it is more valuable to clarify whether there is a paradox in the general employability. In summary, it is of great and irreplaceable importance to explore the paradox of general employability in theory and practice.

In fact, the employability paradox controversy focuses mainly on discussing the impact on negative results, such as turnover, and there are other ways to address why various results have been found in existing research. Recent studies have found that perceived employability and turnover may be positively correlated (De Cuyper et al., 2011a,b; Virga et al., 2017), uncorrelated (Berntson et al., 2010), or negatively correlated based on moderating variables (Acikgoz et al., 2016). We believe that these contradictory linear results may be due to the inverted U-shaped non-linear relationship between variables (Pierce and Aguinis, 2013). In recent years, non-linear models have gradually received attention in the OB field (Grant and Schwartz, 2011). Some researchers have suggested that in the future, we should analyze the influence of social/interpersonal compatibility on employability from both aspects of linearity and non-linearity (Ferris and Summers, 2013). This possibility inspired us to consider the relationship between perceived employability and turnover intention from a non-linear perspective.

### Relationship Between Perceived Employability and Turnover Intention

We rely on two theories to formulate our hypotheses: the COR theory and prospect theory. The COR theory is a motivation theory with basic tenet that individuals (and groups) strive to obtain, retain, foster, and protect valuable resources (Hobfoll et al., 2018). The prospect theory finds that when people face decision-making, they do not make completely rational calculations of the expected utility based on the risk and return of the choices. In actual decision-making, people’s perceptions of risk and the final decision results often deviate from the optimal decision result of traditional economic theory (Kahneman and Tversky, 1979). Specifically, individuals show tendencies toward risk aversion when facing gains and toward risk seeking when facing losses (Kahneman and Tversky, 1979). Both COR and prospect theory focuses on individual’s attitude and reaction to gains and losses. The COR theory points out that increasing resource gain and reducing resource loss are the motivation of human behaviors. The prospect theory indicates that in the face of gain and loss, individuals perform different risk-aversion/seeking decisions (Shimizu, 2007). Combining these two theories, the relationship between perceived employability and turnover intention can be explained more comprehensively.

When employees want to leave, in addition to considering the actual opportunities in the labor market, they will ask two questions: a. Can I find a better job soon after leaving? b. Do I want to leave? When any answer is “No,” then their turnover intention will be very low. Therefore, the level of turnover intention will be affected by both ability and motivation. From the perspective of ability, for employees with low perceived employability, they feel it would be difficult to find more or better job opportunities. Therefore, the perceived ability to leave (job-hop) is low. Employees with high perceived employability are just the opposite. Therefore, from the perspective of ability, the higher the perceived employability is, the higher the perceived ability to leave is. Second, from the perspective of motivation, the turnover behavior will not only bring about the resource losses contained in the existing work but also bring new resource gain because of finding a new job. For individuals with low perceived employability, leaving means a loss of current resources, and low perceived employability makes it difficult for them to find a new or better job. Therefore, the comprehensive resource benefits brought by turnover behaviors are likely to be negative (i.e., losing the existing job but unlikely to find a new and better job), that is, losing resources. Therefore, these people will not be likely to leave from the COR perspective. For individuals with high perceived employability, they feel more likely to find more and better job opportunities; therefore, the benefits of new jobs will be higher than the loss of leaving the existing job. Therefore, the total benefits of turnover behavior will be positive in total (losing the existing job but very likely to find a better new job), i.e., profitable. For them, the turnover behavior is a potential resource gain from the COR perspective. However, based on the prospect theory, people will show a risk aversion tendency when facing a potential gain situation. The more current resources they have, the more risk-averse they become. Therefore, individuals with high perceived employability may also be reluctant to leave. Considering both the ability and motivation, individuals with low perceived employability have low motivation and abilities to quit. Individuals with high perceived employability have high abilities but low motivations, which also results in low turnover intention. However, when the perceived employability is moderate, the individual’s ability and motivation of turnover are both at an intermediate level, which will cause a higher turnover intention than both those with lower or higher perceived employability, because the interaction effect of both ability and motivation are higher. That is, with improvement in perceived employability, the interaction between an employee’s perceived turnover ability and motivation will lead to a trend of turnover intentions increasing from low to high and then decreasing to a low level, showing the characteristics of an inverted U-shaped relationship.

A more generalized and systematic explanation of the inverted U is the “too much of a good thing effect” (TMGT effect) (Pierce and Aguinis, 2013). For turnover intentions, perceived employability is generally regarded as a positive influencing factor. The TMGT effect accounts for a paradox by which ordinarily beneficial antecedents cause harm when taken too far (Pierce and Aguinis, 2013); it indicates that seemingly positive relations reach some points after which the relations turn unexpectedly negative, resulting in an overall inverted U-shaped curve (Grant and Schwartz, 2011). A common reason for the TMGT effect is the added benefit and cost (Haans et al., 2016). As the independent variable increases, with the marginal cost for dependent variable increasing and marginal benefits diminishing, an inverted U-shaped relationship occurs (Grant and Schwartz, 2011). As discussed previously, turnover will bring both resource loss and gain. According the corollary 1 of COR theory, those with greater resources are more capable of gaining resource. As a personal resource (De Cuyper et al., 2012), people with higher perceived employability will have more resources. When perceived employability increases, both the loss of current job and the gain of a new job associated with turnover will also increase. The principle 1 of COR theory, i.e., primacy of loss principle, indicates that resource loss is disproportionately more salient than resource gain, from which we can infer that the marginal cost caused by the turnover’s resources loss will increase when the perceived employability increases, whereas the marginal benefit caused by the gain will decrease. Specifically, the increase in perceived employability will bring about an increase in various resources, resulting in a reduction in the sensitivity of individuals to resource gain, so the marginal benefit decreases. However, with the accumulation of resources by working hard in an organization for years, turnover will bring a rapid loss of current resources, especially for those with high perceived employability who have already obtained long-term incentives, such as employee stock ownership plans. Therefore, the marginal cost of turnover increases as perceived employability increases. In conclusion, the increase in perceived employability will lead individuals to expect that turnover behavior will bring marginal benefit decrease and marginal cost increase; therefore, the superposition effect will cause the turnover intention to show an inverted U-shaped relationship. Accordingly, we propose the following assumption:

Hypothesis 1a: There is an inverted U-shaped relationship between perceived employability and turnover intention.

### Relationship Between Perceived Employability and Job Performance

The relationship with job performance is an important function that has attracted considerable attention to perceived employability (Van der Heijde and Van der Heijden, 2006). As mentioned previously, the COR theory proposes that highly resourceful individuals can gain more resources more easily. Therefore, employees with high perceived employability are more likely to obtain more performance-supporting resources, such as mastering better industry information or higher-level LMX, which will make them more capable and motivated to achieve high performance. The resource caravans principle of the COR theory also posits that resources do not exist in isolation. Many resources exist as if they were sitting in a caravan because they are generated in a similar environment and are highly related (Hobfoll et al., 2018). It can be inferred that perceived employability and other resources that support high performance usually exist at the same time. Considerable existing research provides strong support for the positive correlation between perceived employability and job performance (De Cuyper et al., 2011a; Bozionelos et al., 2016; Hennekam, 2017). For the completeness of testing employability paradox, we test the perceived employability-performance relationship, even though this relationship is not the focus of controversy and the current study. We hypothesize as follows:

Hypothesis 1b: There is a positive correlation between perceived employability and job performance.

### Seniority and Its Impact on Employability Paradox

The test of the employability paradox also needs to consider relevant contextual contingency factors (Nelissen et al., 2017). Age is an important factor in career development (Van der Heijden et al., 2009; De Vos et al., 2017; Van der Heijde et al., 2018; Le Blanc et al., 2019). However, for in-service employees, the overall time of employment (i.e., seniority) is more closely related to employment. Compared with age, employee seniority is a more direct and pure related factor to the employment status because it excludes the interference of different education years from age. Baker and Eaton’s (1992) quasi-experimental study confirmed that seniority is a more important factor than physical age in affecting employees’ value to the team. Hobfoll et al. (2018) regarded seniority as a condition resource. We conclude that seniority may interact with perceived employability (also a resource) to influence employee behavior. The reasons are as follows.

First, new employers will have different attitudes toward job-hopping behaviors by candidates with different seniority and perceived employability which, in turn, will affect employees’ willingness to change jobs. The theory of career timetable points out that organizations have different attitudes and management principles for employees of different ages (Lawrence, 1984). It can be inferred that organizations have different expectations for employees of different seniority. For employees with shorter seniority, organizations usually have a relatively positive attitude toward the phenomenon that employees with high perceived employability have a higher turnover intention because it can be regarded as a way of “finding themselves” or “ambition.” For those with longer seniority, employees with high perceived employability usually perform well and work stably. If they leave, they may receive negative attributions, such as “cannot persist in a job” or “difficult to adapt” (Lam et al., 2012). Similarly, new employers will have similar attributions to candidates with different seniority and perceived employability. This attitude will directly affect the success rate of employees’ job hopping, which is an important factor for employees considering whether to leave. Therefore, as a condition resource, seniority will also affect the employees’ turnover intention together with perceived employability.

Second, according to the career timetable theory, individuals may take different routes to achieve career success at different stages of seniority because individuals are expected to have a different sequence of actions in their lives (Lawrence, 1984). Thus, employees’ turnover and performance will also vary. Employees in the early career stage rely on job transfer to obtain higher human and social capital, whereas employees in their middle or late career stages will have reduced ability and desire to switch employers (Lam et al., 2012) and are more likely to rely on higher job performance in a current position to increase job competitiveness. Therefore, in order to achieve career success, people with shorter seniority tend to rely on their employability to find new job opportunities, so during this time, perceived employability has a crucial impact on turnover intentions. Employees with longer seniority have relatively stable jobs, have reached a certain level of career identity, and are more inclined to achieve career success by achieving better work performance, rather than changing jobs.

Therefore, we believe that the perceived employability of employees with shorter seniority has a greater impact on turnover intentions, whereas the perceived employability of employees with longer seniority has a greater impact on job performance.

In China, employers and researchers usually consider 3 years’ seniority as a threshold for a mature or experienced employee. Allen and Meyer (1993) regard 2 years and less as the first stage of seniority, but in terms of Chinese employment reality, most employers set 3 years as the first contract period when signing labor contracts. Three years’ seniority is an important dividing line in Chinese employment practice. Therefore, this article divides the seniority into a shorter period less than or equal to 3 years and a longer period of more than 3 years. We assume the following:

Hypothesis 2a: The relationship of perceived employability with turnover intention will be moderated by the employee’s seniority. The relationship will be stronger for employees with shorter seniority (≤3 years) than for those with longer seniority (>3 years).
Hypothesis 2b: The relationship of perceived employability with job performance will be moderated by the employee’s seniority. The relationship will be stronger for employees with longer seniority (> 3 years) than for those with shorter seniority (≤3 years).

## Materials and Methods

### Participants

Based on management consulting projects, MPA training projects and personal social networks, surveys were collected in China from employees and their managers in 27 enterprises in the cities of Beijing, Guangzhou, Shenzhen, Zhengzhou, Nanchang, Tai’an, and Xuchang in two waves. After obtaining top managers’ support, researchers organized employees to fill in the questioners in their workplace. Employees were asked to mark their employee ID on the envelope, with the notification of researchers that the survey were only for academic research. HR managers were responsible for the collection of manager-rated questioners. Each questioner was marked with relevant employee ID. Employees and their manager were matched through the employee ID by researchers. Employees were asked to complete the employability questionnaire at Time1 and their turnover intention two weeks later (Time2). Job performance was evaluated by their direct managers at Time2. A total of 1,100 questionnaires were distributed at two time points. From these, 623 matched employee and manager surveys were identified, generating a response rate of 56.6%. Details for the enterprises and employees are summarized as follows: organization size (300 employees and below 38.4%, 300–500 employees 20.1%, above 500 employees 41.6%); education level (junior college degree and below 57.1%, bachelor’s degree and above 42.2%, missing 0.6%); gender (male 42.9%, female 57.0%, missing 0.2%); contract type (permanent 86.2%, temporary 12.5%, missing 1.3%); native place (countryside 60.5%, city 39.5%); and work seniority (3 years and below 71.3%, above 3 years 27.4%, missing 1.3%).

### Measures

Because the original questionnaire used in the survey was developed in English, translation and back-translation procedures were adopted to convert it to Chinese.

*Perceived employability* was measured by a 4-item scale developed by De Cuyper et al. (2011a). A sample item is: “I will easily find another job if I lose this job.” Employees were asked to rate on a 5-point Likert scale, where 1 = strongly disagree, and 5 = strongly agree. The Cronbach’s alpha was 0.85 in this study.

*Turnover intention* was measured by a 3-item scale developed by Konovsky and Cropanzano (1991). A sample item is: “How likely is it that you will look for a job outside of this organization during the next year?” Employees were asked to rate, and the 5-point scale depends on the question wording, as for the sample item, 1 = very unlikely, and 5 = very likely. The Cronbach’s alpha was 0.86 in present study.

*Job performance* was measured using a 3-item scale developed by Motowidlo and Van Scotter (1994). It was measured by three 5-point scales, each with a different set of anchors at the high, moderate, and low ranges. A sample item and scale is: high (4–5) = exceeds job performance standards, moderate (3) = meets job performance standards, and low (1–2) = fails to meet job performance standards. In this study, the Cronbach’s alpha was 0.89.

Demographic variables including gender, contract type, education level, and native place were selected as control variables.

### Data Analyses

First, we adopted SPSS 18.0 to analyze the descriptive statistics, correlations, and internal consistency reliabilities of the variables. Second, Mplus 7.4 was used to conduct confirmatory factor analysis to examine the discriminant validity. Also, by using data resulting from Mplus, we calculated the average variance extracted and composite reliability with Excel. Third, common method variance was tested by exploratory factor analysis with SPSS. Fourth, hierarchical regression analyses were conducted for the hypotheses testing with SPSS. In this step, in order to reduce the possible multicollinearity caused by the construction of the square term, the square term of perceived employability in the regression analysis was calculated by centralizing and then squaring.

## Results

### Descriptive Statistics and Preliminary Analyses

The variables’ means, standard deviations, correlations, and internal consistency reliabilities are presented in Table 1.

**TABLE 1:** Means, standard deviations, correlations, and internal consistency reliabilities.

**Variable**	***M***	**SD**	**AVE**	**CR**	**1**	**2**	**3**	**4**	**5**	**6**	**7**	**8**	**9**
1. Gender	1.57	0.50	–	–	–								
2. Contract type	1.13	0.33	–	–	0.07	–							
3. Education level	1.42	0.49	–	–	–0.05	–0.04	–						
4. Native place	1.39	0.49	–	–	0.13**	0.01	0.18**	–					
5. Work seniority	1.28	0.45	–	–	–0.02	0.03	0.21**	0.11**	–				
6. Perceived employability	3.53	0.83	0.59	0.85	0.00	−0.09*	0.18**	0.06	–0.00	0.85			
7. Perceived employability^2^	0.69	0.96	–	–	–0.09*	–0.00	–0.10*	–0.03	–0.01	0.10*	–		
8. Turnover intention	2.67	1.05	0.68	0.87	0.05	0.00	–0.06	0.07	0.11**	–0.00	−0.16**	0.86	
9. Job performance	3.41	0.73	0.72	0.89	0.09*	–0.04	0.09*	0.16**	0.16**	0.04	–0.03	0.12**	0.89

*p < 0.05, **p < 0.01; gender, 1 = male,2 = female; contract type, 1 = permanent, 2 = temporary; education level, 1 = junior college and below, 2 = bachelor’s degree and above; native place, 1 = countryside, 2 = city; work seniority, 1 = 3 years and below, 2 = above 3 years; α-reliability coefficients are on the diagonal.

A confirmatory factor analysis was conducted to test the measurement model’s validity with three target variables. The results analyzed by Mplus 7.4 showed the 3-factor model (perceived employability, turnover intention and job performance) had the best fit, with χ^2^/df = 1.717, CFI = 0.938, TLI = 0.989, RMSEA = 0.034, and SRMR = 0.027, indicating the three constructs are independent and clear and have good discriminant validity. In addition, following Fornell and Larcker (1981), the variables were examined for convergent validity by estimating whether the scale’s average variance extracted (AVE) is above 0.5 and composite reliability (CR) is above 0.7. The results presented in Table 1 indicate all scales in this study have acceptable validity.

Two methods suggested by Podsakoff et al. (2003) were adopted to control common method variance. First, two techniques of different time points and multiple data sources were used in conducting the surveys: employees evaluated their employability at time1 and turnover intention 2 weeks later at time2, and the direct managers also evaluated their job performance at time 2. Second, common method variance was tested by adopting Harman’s single-factor test and an exploratory factor analysis. The results showed that the first factor drawn from exploratory factor analysis only accounted for 28.35% of the variance (overall variance accountability is 76.08%). No common factor was found in unrotated factorial structure. The confirmatory factor analysis results also showed that the RMSEA (0.304) of the single-factor model did not meet the psychometric criterion (Bollen, 1989), indicating the inexistence of a common factor. Thus, common method variance was not a significant problem in this study.

### Hypotheses Testing

Hierarchical regression analysis by SPSS 18.0 was used to test the hypothesis. The results (see Table 2) show no significant linear correlation between perceived employability and turnover intention (M2 in Table 2, β = −0.00, *p* > 0.05). However, there is an inverted U-shaped relationship between perceived employability and turnover intention (M3 in Table 2, β = −0.15, *p* < 0.01); therefore, Hypothesis 1a is supported. The results also show no significant relationship between perceived employability and job performance (M8 in Table 2, β = 0.02, *p* > 0.05); therefore, Hypothesis 1b is not supported.

**TABLE 2:** Regression results for turnover intention and job performance (*N* = 623).

**Variables**	**Turnover intention**						**Job performance**			
**Models**	**M1**	**M2**	**M3**	**M4**	**M5**	**M6**	**M7**	**M8**	**M9**	**M10**
Gender	0.04	0.04	0.03	0.03	0.03	0.03	0.07	0.07	0.07	0.07
Contract type	–0.00	–0.00	–0.00	–0.01	–0.01	–0.01	–0.06	–0.06	–0.07	–0.07
Education level	–0.07	–0.07	–0.05	–0.08	–0.07	–0.07	0.08	0.08	0.05	0.05
Native place	0.07	0.07	0.06	0.06	0.06	0.05	0.14**	0.14**	0.13**	0.13**
Perceived employability		–0.00	–0.02	–0.14	–0.01	–0.02		0.02	0.02	0.02
Perceived employability^2^			−0.15**	−0.15**	−0.15**	−0.14**				
Work seniority				0.11**	0.11**	0.03			0.14**	0.14**
Perceived employability × work seniority					0.06	0.07				0.09*
Perceived employability^2^ × work seniority						0.14**				
R^2^	0.011	0.011	0.034	0.045	0.049	0.061	0.042	0.042	0.060	0.068
△R^2^	0.011	0.000	0.023**	0.011**	0.004	0.012**	0.042	0.000	0.019**	0.008*

*p < 0.05, **p < 0.01; gender, 1 = male,2 = female; contract type, 1 = permanent, 2 = temporary; education level, 1 = junior college and below, 2 = bachelor’s degree and above; native place, 1 = countryside, 2 = city; work seniority, 1 = 3 years and below, 2 = above 3 years.

**FIGURE 1:**
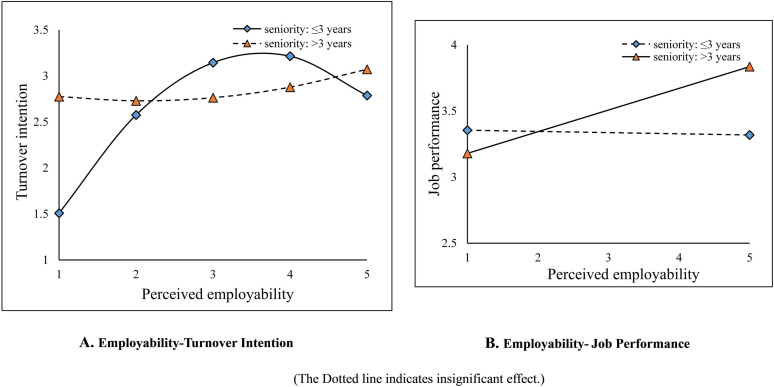
Effects of work seniority on perceived employability-turnover intention, and perceived employability-job performance relationship.

Table 2 shows seniority’s moderating effect on the relationship between perceived employability and turnover intention. The interaction term of perceived employability square and seniority has a significant effect on turnover intention (M6 in Table 2, β = 0.14, *p* < 0.01). Table 3 (M1–M6) further illustrates seniority’s moderating effect. The perceived employability and turnover intention of employees with shorter seniority (≤3 years) shows an inverted U-shaped curve (β = −0.22, *p* < 0.01), whereas for employees with longer seniority (>3 years), the relationship disappears (linear: β = 0.10, *p* > 0.05; non-linear: β = 0.04, *p* > 0.05). Therefore, Hypothesis 2a is supported. Figure 1A shows the relationship between perceived employability and turnover intention for employees with different seniority.

**TABLE 3:** Effects of work seniority on perceived employability-turnover intention and perceived employability-job performance relationship.

**Variables**	**Turnover intention**						**Job performance**			
**Seniority**	**≤3 years**			**>3 years**			**≤3 years**		**>3 years**	
**Models**	**M1**	**M2**	**M3**	**M4**	**M5**	**M6**	**M7**	**M8**	**M9**	**M10**
Gender	0.06	0.06	0.04	–0.03	–0.02	–0.02	0.07	0.07	0.07	0.08
Contract type	–0.07	–0.08	–0.08	0.15*	0.15*	0.15*	–0.06	–0.06	–0.11	–0.10
Education level	−0.13**	−0.12*	–0.09	–0.02	–0.04	–0.04	0.03	0.04	0.11	0.09
Native place	0.01	0.01	0.01	0.18*	0.18*	0.18*	0.13**	0.13**	0.14	0.13
Perceived employability		–0.03	–0.06		0.09	0.10		–0.03		0.18*
Perceived employability^2^			−0.22**			0.04				
R^2^	0.026	0.027	0.072	0.053	0.061	0.063	0.029	0.030	0.061	0.092
△R^2^	0.026	0.001	0.045**	0.053	0.008	0.001	0.029	0.001	0.061	0.031*

*p < 0.05, **p < 0.01; gender, 1 = male,2 = female; contract type, 1 = permanent, 2 = temporary; education level, 1 = junior college and below, 2 = bachelor’s degree and above; native place, 1 = countryside, 2 = city.

Table 2 shows the moderating effect of seniority on the relationship between perceived employability and job performance. The interactive term of perceived employability and seniority has a significant impact on job performance (M10 in Table 2, β = 0.09, *p* < 0.05). Table 3 (M7–M10) further illustrates this moderating effect. For employees with shorter seniority (≤3 years), the relationship between perceived employability and job performance is not significant (β = −0.03, *p* > 0.05); for those with longer seniority (>3 years), this relationship is significantly positive (β = 0.18, *p* < 0.05). Figure 1B shows the relationship between perceived employability and job performance for employees of different seniority. Therefore, Hypothesis 2b is supported.

## Discussion

The purpose of this study is to determine whether the employability paradox exists by combining linear and non-linear analysis. This question is answered by examining the relationship between perceived employability, turnover intention and job performance, and a condition variable. The study results reveal the complex relationship between perceived employability and turnover intention. The positive correlation (De Cuyper et al., 2011a,b; Virga et al., 2017), no correlation (Berntson et al., 2010) and negative correlation based on moderators (Acikgoz et al., 2016; Rodrigues et al., 2020) between perceived employability and turnover intention suggest the linear results are controversial. With the combination of COR and prospect theory, we explained the relationship could be positive and then to negative when the level of perceived employability is from low to high. The TMGT effect can also help to interpret. Our findings proved this inverted U-shaped relationship. It broke the limitation of adopting only linear approach. In particular, the promotion of turnover intention mentioned in the employability paradox only occurs when the perceived employability is low (but it does not promote job performance in this case).

Second, the hypothesis of positive correlation between perceived employability and job performance was not confirmed, which is also consistent with some existing literature. This may be due to mediating variables, such as perceived employability and job performance, that are not directly related but can affect job performance through emotional organizational commitment (De Cuyper and De Witte, 2011), or to the influence of moderating variables such as job security (De Cuyper et al., 2014), perceived justice (Philippaers et al., 2019), and the seniority concerned in this study. For example, when employees’ job security is high, perceived employability has no significant impact on job performance. But when employees’ job security is low, the relationship is significantly positive (De Cuyper et al., 2014). This study found no direct impact of employability on job performance. It may be due to the existence of potential mediators and moderators. This finding also highlights the necessity of exploring the relationship under different work seniority from the perspective of career timetable. Furthermore, it can also inspire future studies on involving moderating and mediating variables into the theoretical model to explore the complex mechanism in the employability paradox.

Third, the results confirmed seniority’s moderating effect on the mechanism of perceived employability. This study found that the inverted U-shaped relationship between perceived employability and turnover intention only exists among employees with shorter seniority (≤3 years). For those with longer seniority (>3 years), the perceived employability only affects their work performance. That is, longer seniority can diminish or magnify the employability-turnover intention/performance relationship, respectively.

Overall, we testified that the general employability paradox of both improving performance and promoting turnover intention does not exist. In contrast, when the employee’s perceived employability level is relatively high, improving the perceived employability will instead reduce the intention to quit. Even when considering seniority, we found that this paradox still does not exist because the increase in perceived employability of employees with shorter seniority only affects their intention to leave, showing an inverted U-shaped relationship, and for employees with longer seniority, the increase in perceived employability only increases performance but does not significantly affect their turnover intention.

### Theoretical Contributions and Practical Implications

This study has three theoretical contributions. First, the general employability paradox is discussed from an integrative perspective of both positive and negative. On the one hand, previous studies have mostly explored the employability paradox from a single perspective of positive utility (such as job performance or organizational commitment) or negative utility (such as turnover or job search intensity) to the organization, with less joint consideration (see De Cuyper et al., 2011a; Rodrigues et al., 2020 for exception). This study comprehensively examines both the positive and negative effects. On the other hand, unlike previous studies discussing whether the paradox exists from the perspective of perceived internal and external employability, this study deeply analyzes the general employability paradox. The internal/external perspective cannot explain the mixed findings in the past, whereas this study provides an insightful alternative. Research on internal/external employability indicates that the idea of this paradox is simplistic and lacks theoretical and empirical support (De Cuyper and De Witte, 2011; De Vos et al., 2017; Nelissen et al., 2017; Rodrigues et al., 2020). Our results echo this view and further indicate that the general employability paradox does not exist, because its promotion effect on performance and turnover does not appear at the same time. Only when employees have short seniority (≤3 years) and low perceived employability will the improvement of perceived employability enhance turnover intention. In some cases, the increase in perceived employability will even lead to a decrease in turnover, which is a positive result for the organization.

Second, the idea of a non-linear inverted U-shape integrates the inconsistent research conclusions of the past and enriches the COR theory. On the one hand, the inverted U-shaped test idea responds to various inconsistent results in previous studies, which helps to address the shortcomings of the two approaches (general employability with linearity, internal/external perspective) in previous employability paradox studies, expands our understanding of the impact of perceived employability on turnover intention, and breaks the original thinking of focusing only on a linear relationship. On the other hand, by combining COR theory, prospect theory and the TMGT effect, this study explains why the non-linear relationship exists from the perspective of the additive effect of resource gains and resource losses, thereby enriching the COR theory. It shows that the COR theory has a strong explanatory power for the non-linear relationship when combined with TMGT effect or prospect theory, as well as for explaining the common linear relationship. Meanwhile, the combination of COR and prospect theory can inspire other scholars who focus on the superposition effect of resource gains and resource losses to propose and explain research problems from a more comprehensive theoretical perspective.

Third, from the career timetable perspective, the conditional features of the non-existent employability paradox are examined in depth. This study verifies the effect of perceived employability on performance and turnover intention among employees with different seniority. The results are consistent with the career timetable theory (Lawrence, 1984; Lam et al., 2012). This finding provides a new way to study the boundary conditions of the mechanism of perceived employability and enrich the career timetable theory.

The implications for practice are as follows. First, it shows the positive value of employability in management practice. The results negate employability paradox, which can convince managers to believe the value of improving perceived employability. Second, it helps managers identify who are more suitable for employability investment: employees with high perceived employability. In this way, they prefer to stay in the organization rather than quit. However, if the employability is too low, it will cause a dilemma of more investment and more turnover. It needs to be improved in recruitment and selection, and elimination mechanism. Third, the research puts forward a suitable time table to improve employability: employees with longer seniority (>3 years), so the action can improve work performance without increasing turnover intention; or employees with shorter seniority (≤3 years) and higher perceived employability so it can reduce turnover intention at this time.

### Limitations and Future Extensions

This study’s limitations lie mainly in the following aspects, which can be changed in future research. First, this study explored the paradox from the perspective of general employability. Compared with the internal and external employability perspectives, we explained that this general perspective has important value in both theory and practice. Following our findings, researchers can further explore the influence of internal and external employability on performance and turnover intention with non-linear approaches in the future. Also the polynomial regression and surface response analysis (Edwards, 2002) could be adopted to discuss the effect of congruence and incongruence of internal/external employability. These approaches may contribute to a broader understanding of the employability paradox. Second, this study measured employability only from the perspective of subjective feelings. In the future, researchers can adopt a measurement perspective based on objective factors, such as ability, dispositions, and social capital, to compare the relationship between subjective perception and objective factors based on employability, and the similarities and differences of their respective roles in the same impact model. Third, the division of seniority is somewhat general. In the future, researchers can further subdivide the employees with more than 3 years of seniority to explore the employability paradox. In particular, attention should be paid to employees in the middle and late career stages. For example, De Vos et al. (2017) divided three different age groups for research by taking 35 and 50 years old as boundaries. Fourth, this study demonstrates the complexity of perceived employability affecting organizational results. Some mediating variables such as organizational commitment (De Cuyper and De Witte, 2011) may also be considered in the future. More contextual factors should be considered to reveal the specific conditions under which paradoxes may occur or not. For example, job security (De Cuyper et al., 2014) and perceived justice (Philippaers et al., 2019) have been proved to be conditional variables of perceived employability influencing performance. Job control (De Cuyper et al., 2011b), protein career orientation (Rodrigues et al., 2020) and tenure (Acikgoz et al., 2016) are potential conditional variables that affect the employability-turnover intention relationship. In addition to perceived employability as a resource, a meta-analysis found that workplace resources at the individual, the group, the leader, and the organizational levels were all related to performance (Nielsen et al., 2017). In the future, researchers can combine COR and prospect theory relevant with resource gains and losses to explore the paradox more widely. Fifth, this study focuses on the turnover intention to discuss the negative side of the employability paradox. However, there are still differences between perceived turnover intention and actual turnover behavior (Vardaman et al., 2015). In the future, a perceived employability- actual turnover behavior based paradox should be adopted (Nelissen et al., 2017), with a cross cultural perspective suggested by Wong and Cheng (2020). Sixth, including this study, empirical findings on employability paradox were all limited in single-level significant models so far (see De Cuyper et al., 2011a; De Cuyper and De Witte, 2011; Acikgoz et al., 2016; De Vos et al., 2017; Nelissen et al., 2017; Imam and Chambel, 2020; Rodrigues et al., 2020). In the future, researchers could take high level factors into account, such as organization and team, to formulate a multilevel structure model of employability paradox. Seventh, the survey was conducted in two waves, and we set 2 weeks as the duration to reduce common method bias. It’s a relatively short period so as to decrease the possibility of occurring events that might affect perceived employability. However, it is still possible that they have really happened. In the future, researchers should control possible events such as formal on-the-job training and upward job transition (Nelissen et al., 2017) during the gap of data collections. Also, a longitudinal approach is suggested to track and test perceived employability’s effects.

## Conclusion

The findings suggest that the employability paradox is invalid because perceived employability is not significantly related to job performance in general and has an inverted U-shaped relationship with turnover intention. In addition, the employees’ seniority is also an important factor that affects the employability paradox. The perceived employability of employees with shorter seniority (≤3 years) affects only turnover intention through an inverted U-shaped relationship but has no impact on performance, whereas for employees with more than 3 years of seniority, perceived employability only promotes performance but does not affect turnover intention; therefore, the paradox still does not exist.

## Data Availability Statement

The raw data supporting the conclusions of this article will be made available by the authors, without undue reservation.

## Ethics Statement

Ethics review and approval were not required for the study on human participants in accordance with the local legislation and institutional requirements. Also written informed consent from the participants was not required to participate in this study in accordance with the national legislation and the institutional requirements.

## Author Contributions

HY carried out the study conception, structure, and data acquisition. CY carried out the analysis and interpretation of data. HY and CY carried out the drafting of the manuscript. HY, CY, LZ, and ZD contributed in critical revision. All authors participated in the discussion of the results and manuscript editing.

## Conflict of Interest

The authors declare that the research was conducted in the absence of any commercial or financial relationships that could be construed as a potential conflict of interest.

## Publisher’s Note

All claims expressed in this article are solely those of the authors and do not necessarily represent those of their affiliated organizations, or those of the publisher, the editors and the reviewers. Any product that may be evaluated in this article, or claim that may be made by its manufacturer, is not guaranteed or endorsed by the publisher.
